# Effects of Formulation and Process Variables on Gastroretentive Floating Tablets with A High-Dose Soluble Drug and Experimental Design Approach

**DOI:** 10.3390/pharmaceutics10030161

**Published:** 2018-09-17

**Authors:** Prakash Thapa, Seong Hoon Jeong

**Affiliations:** College of Pharmacy, Dongguk University-Seoul, Gyeonggi 10326, Korea; thapap139@gmail.com

**Keywords:** polyethylene oxide, sodium bicarbonate, gel strength, floating lag time, drug release kinetics, experimental design

## Abstract

To develop sustained release gastro-retentive effervescent floating tablets (EFT), a quality-based experimental design approach was utilized during the composing of a hydrophilic matrix loaded with a high amount of a highly water-soluble model drug, metformin HCl. Effects of the amount of polyethylene oxide WSR 303 (PEO), sodium bicarbonate, and tablet compression force were used as independent variables. Various times required to release the drug, tablet tensile strength, floating lag time, tablet ejection force, and tablet porosity, were selected as the responses. Polymer screening showed that PEO had the highest gel strength among the various tested polymers. Sodium bicarbonate had the most significant effect on the release rate and floating lag time by retarding the rate from the hydrophilic matrices, whilst tablet compression force and PEO exerted the greatest influence on tablet properties (*p* < 0.0001). The design space was built in accordance with the drug release profiles, tensile strength, and floating lag time, following failure probability analysis using Monte Carlo simulations. The kinetic modeling revealed that the release mechanism was best described by the Korsmeyer-Peppas model. Overall, the current study provided a perspective on the systematic approach of gastro-retentive EFT, loaded with highly water-soluble drugs by applying quality by design concepts.

## 1. Introduction

Even though oral drug delivery is the most convenient and preferable route of drug administration, there are still challenges to overcome. Bioavailability of active pharmaceutical ingredients (APIs) is subject to change, depending on their physicochemical properties, including pH-dependent solubility and stability, and a narrow absorption window [1,2,3]. Therefore, formulation scientists are continuously engaged in developing new approaches to improve oral drug delivery systems. Recently, gastroretentive systems, with strategies to extend gastric residence time, have drawn considerable attention as an alternative approach to improve bioavailability of drugs with a narrow absorption window, stability at intestinal pH, local activity in the stomach, and solubility at low pH [4,5,6]. The gastric residence time can affect the drug absorption, as the longer the drug stays in contact with the absorbing membrane, the more the rate and extent of absorption [3,7]. However, residence time in the upper part of the gastrointestinal tract is short due to fast gastric emptying lasting about 2–3 h [3,8]. To overcome the limitation, controlled drug delivery systems with prolonged residence time in the stomach can be utilized.

Various pharmaceutical approaches have been applied to prolong the gastric retention time of dosage forms, including floating systems, bioadhesive/mucoadhesive systems, expandable systems, high density systems, superporous hydrogel systems, and magnetic systems [6,9,10,11,12]. However, among the gastroretentive systems, floating systems offer promising and practical means of achieving prolong gastric residence time [2,5,6,8,12]. The floating systems are categorized into non-effervescent systems and effervescent systems. In the case of non-effervescent systems, highly swellable cellulose derivatives or gel-forming polymers are used, which is preferable for potent drugs [13,14,15]. However, in effervescent systems, gas generating agents, such as sodium bicarbonate and calcium carbonate are used, which generate CO_2_ gas upon contact with gastric fluid, and eventually reduce the bulk density of tablets [16,17,18,19].

It is often challenging to maintain tablet buoyancy in high-dose tablets due to their high bulk density; therefore, the non-effervescent technique may not be feasible for such tablets. Effervescent floating tablets (EFTs) have better potential for improved buoyancy [20,21,22]. The selection of a suitable polymer, a gas generating agent, and process variables might be necessary for high-quality EFT development. A large amount of polymer is required to achieve sustained release profiles in highly water-soluble high-dose tablets, which in turn increases the weight of tablets [21,23,24,25]. Hydrophilic polymers have long been used in floating systems to create sustained release profiles. Among various hydrophilic polymer types (cationic, anionic, and non-ionic polymer), the non-ionic hydrophilic polymers, such as hydroxypropyl methylcellulose (HPMC), hydroxypropyl cellulose (HPC), and polyethylene oxide (PEO), are commonly used in controlled release tablets because these polymers are not affected by pH [13,26]. As a result, drug release and floating behavior of tablets are also not influenced by the pH of gastric fluid [13,26]. In addition, non-ionic hydrophilic polymers are non-toxic, economic, and safe to use for EFT [27,28,29]. Therefore, these types of polymers have potential in designing the floating tablet. Moreover, other factors, including amount of polymer and polymer viscosity grade and molecular weight may influence the drug release rate and tablet buoyancy, as well as other physicochemical properties, such as tablet tensile strength, porosity, hydration rate, and gel strength [30].

In the effervescent system, sodium bicarbonate improves tablet buoyancy in gastroretentive floating systems containing hydrophilic polymer combinations. However, no systematic investigation has yet been performed to explore the influence of sodium bicarbonate on drug release kinetics. Therefore, it is important to investigate its influence on the drug release kinetics of highly water-soluble drugs in the EFTs. From the perspective of pharmaceutical technology, tablet compression force has the potential to affect tablet density and this could alter tablet buoyancy, as tablet density >1.004 mg/cm^3^ prevents floating in the gastric fluid [3]. Therefore, optimization of tablet compression force might overcome issues of table buoyancy, as well as friability and mechanical properties. In the present study, we also investigated the impact of compression force on tablet buoyancy and other physiochemical properties.

Formulation scientists often experience the challenges of identifying the appropriate combination of formulation and process variables to produce a high-quality product [13,31,32,33]. However, with the application of quality-based experimental design tools, the variables can be more easily analyzed and understood. Among the various experimental design tools, Box-Behnken design (BBD) is a popular tool for formulation and process optimization, which utilizes the treatment combination at midpoint of the edge and center of the experimental space. The main advantage of BBD, compared to central composite design (CCD), D-optimal design, and 3-level factorial design, is that it requires fewer experimental runs, less time for optimization of the process, and is more cost effective. In addition, BBD does not have axial points and it can assure that all the design points fall within safe operating zones, whereas CCD usually has axial points outside the cube, which may not be in the region of interest, or may be beyond safe operating limits [33,34]. In the present study, a Box-Behnken design was used to study the impact of formulation variables (PEO, sodium bicarbonate) and a process variable (compression force) on response variables, including drug release rate, floating lag time (FLT), tablet tensile strength, tablet porosity, and tablet ejection force. In addition, other physical properties, such as medium uptake (swelling ratio), tablet erosion rate, and gel strength of floating tablets, were also investigated.

## 2. Materials and Methods

### 2.1. Materials

The model drug, metformin HCl, was obtained from Farmhispania (Catalonia, Spain). Hydroxypropyl methylcellulose (Hypromellose USP, substitution type 2208), HPMC 100SR (viscosity 100 mPa.s), HPMC 4,000SR (viscosity 4000 mPa.s), and HPMC 100,000SR (viscosity 100,000 mPa.s) were obtained from Shin-Etsu (Tokyo, Japan). Hydroxypropyl cellulose, HPC M (viscosity 300 mPa·s), and HPC H FP (viscosity 3000 mPa·s) were supplied from Nippon Soda Co., Ltd. (Tokyo, Japan). Polyethyleneoxide WSR 303 (PEO, average molecular weight 7,000,000) was purchased from Dow Chemical (Midland, MI, USA). Lactose monohydrate (FlowLac 100) was obtained from Meggle Pharma Tech., Ltd. (Wasserburg, Germany), microcrystalline cellulose (MCC, Avicel PH 101) was supplied from Daejung Pharmaceuticals. (Seoul, Korea), dicalcium phosphate anhydrous (A-Tab) was purchased from Whawon Co. Ltd. (Seoul, Korea), and sodium bicarbonate was purchased from Sigma-Aldrich (St. Louis, MO, USA). Magnesium stearate (S-Mg) was obtained from Faci Asia (Jurong Island, Singapore).

### 2.2. Screening of Hydrophilic Polymers

To select the polymer matrix for the floating tablets, gel strength of six different hydrophilic polymers viz. HPC M, HPC H FP, HPMC 100SR, HPMC 4000SR, HPMC 100,000SR, and PEO were evaluated using a texture analyzer. Then, 400 mg of each polymer was added to 10 mL of deionized water in a glass vial and stirred in a water bath at 37 °C, until the polymer was completely hydrated. Samples of hydrated or swollen polymer were stored at room temperature for 24 h, prior to analysis [35,36]. The gel strength was measured using a texture analyzer (TA.XT Express, Stable Micro Systems, Surrey, UK). An analytical probe of 10 mm diameter was penetrated into the hydrated sample to a depth of 8 mm, at speed of 0.5 mm/s [35,36]. Each experiment was performed in triplicate.

### 2.3. Box-Behnken Experimental Design

A BBD with three factors, three levels, and 15 runs was selected for the characterization and optimization (Table 1). The amount of PEO per tablet (*X*_1_), the amount of sodium bicarbonate per tablet (*X*_2_), and tablet compression force (*X*_3_) were selected as input variables, whilst the time taken to release 25% of drug (T25%, *Y*_1_), 50% of drug (T50%, *Y*_2_), 80% of drug (T80%, *Y*_3_), FLT (*Y*_4_), tablet ejection force (EF, *Y*_5_), tablet tensile strength (*Y*_6_), and tablet porosity (*Y*_7_) were selected as dependent variables. Statistical analysis and optimization were carried out using MODDE^®^ software, version 12.0.1 (Sartorius Stedim biotech, Malmö, Sweden). The effects of input variables could be described mathematically, and the response variables could be predicted for each set value of input variables. The non-linear quadratic equation generated using the experimental design was as follows [37]:

(1) $$Y_{i}=b_{0}+\sum b_{i}X_{i}+\sum b_{ii}X_{ii}^{2}+\sum b_{ij}X_{i}X_{j}$$

where, $Y_{i}$ is the responses, $X_{i}$ and $X_{j}$ are the independent variables; $b_{0}$ is a constant term, and $b_{i}$, $b_{ii}$, and $b_{ij}$ are the coefficients of the linear, quadratic, and interaction terms, respectively.

### 2.4. Preparation of Floating Tablets

Metformin, lactose monohydrate, and MCC were passed through a #30 mesh sieve, and PEO was passed through a #20 mesh sieve to remove any aggregates. Sodium bicarbonate was milled in a mortar and pestle for 10 min and passed through a #40 mesh sieve. Metformin and the excipients except S-Mg were mixed using the motor and pestle for 10 min, to obtain a well-dispersed mixture. S-Mg (sieved through a #40 mesh sieve) was added to the above mixture, and then blended for 5 min. Then, 1000 mg of the mixture was loaded into a die and compressed on a hydraulic laboratory press (Carver Press, Wabash, IN, USA) using a 13 mm set of flat face punches at different compression forces, as shown in Table 1. The dwell time for each tablet compression was 5 s. To compare the drug release profiles of tablets containing sodium bicarbonate, tablets without sodium bicarbonate were also prepared. F0 represents the formulation with a low amount of polymer, i.e., 50 mg PEO and 90 mg sodium bicarbonate. Likewise, F2 and F15 contained 100 mg and 200 mg PEO, respectively, and 90 mg of sodium bicarbonate in each formulation. Moreover, additional formulations of F0*, F2*, and F15* without sodium bicarbonate were also prepared. They contained various PEO amounts of 50 mg, 100 mg, and 200 mg, respectively.

### 2.5. In vitro Drug Release Studies

In vitro dissolution tests were conducted according to the US Pharmacopeia dissolution apparatus 2 (paddle method with a paddle speed of 50 rpm), with 900 mL dissolution medium of simulated gastric fluid (pH 1.2), maintained at 37 ± 0.5 °C (Varian 705 DS, Varian, Cary, NC, USA). The tablet was placed in the stationary basket to prevent the tablet from floating or sticking to the inner surface of dissolution vessels. Samples were withdrawn at predetermined time intervals (0.5, 1, 2, 3, 4, 6, 8, 10, and 12 h) from the dissolution vessels, and then replaced with the fresh medium. The aliquots were filtered through a 0.45 µm membrane filter, suitably diluted, and analyzed with a UV spectrophotometer at a wavelength of 233 nm to determine the amount of metformin released over time. The percentage drug released (%), was calculated and provided as the mean value of four tablets.

### 2.6. In Vitro Floating Behavior

Floating behavior of the tablets was determined in similar conditions as those used in the in vitro drug release studies. Time required for the tablets to rise on the surface of the medium was considered as floating lag time (FLT), and the total duration of tablet floating on the medium was considered as floating time. Each experiment was conducted in triplicate.

### 2.7. Medium Uptake and Mass Loss of Tablets

Medium uptake and mass loss of the prepared matrix tablets were evaluated using the dissolution apparatus. Briefly, a pre-weighed tablet $\left(W_{1}\right)$ was transferred into 900 mL of the simulated gastric fluid (pH 1.2, 37.0 ± 0.5 °C), at a rotation speed of 50 rpm. At predetermined time intervals, the swollen tablet was removed from the medium and blotted with a tissue paper to remove the excess medium on the tablet surface, and weighed $\left(W_{2}\right)$ in an analytical balance [24,25,38,39]. Then, the swollen tablet was dried in an oven at 50 °C, until a constant weight was obtained $\left(W_{3}\right)$. Each experiment was performed in triplicate. The percentage of medium uptake and mass loss of the tablet were calculated using the following Equations (2) and (3), respectively.

(2) $$\%\;Medium\;Uptake=\frac{W_{2}-W_{1}}{W_{1}}\times 100\%$$

(3) $$\%\;Mass\;Loss=\frac{W_{1}-W_{3}}{W_{1}}\times 100\%$$

### 2.8. Total Fwork of Penetration

Total work of penetration profiles can give useful information about the gel strength of the hydrated tablet [40,41]. Therefore, mechanical gel strength of the hydrated tablets was evaluated using the texture analyzer equipped with a 5 kg load cell and the software texture expert. The tablets were placed in dissolution vessels under similar conditions as in the in vitro drug release study. The hydrated tablets were removed at different time points, patted lightly with a tissue paper, and subjected to texture profiling, to determine the total work of penetration [15,38,42]. Force-displacement profiles associated with the penetration of a 2 mm round-tipped steel probe into the swollen tablet were monitored [15,38,41,43,44]. All measurements were carried out in triplicate at each time point. When the trigger force reached 0.001 N, signal recording was initiated, and the probe was advanced into the sample at a speed of 0.5 mm/s, until the maximum force of 60 N was attained. The total work of penetration, which is a measure of gel strength and resistance to probe penetration, was determined from textural profiles. In Equation (4), *F* is applied force and *D* is distance travelled.

(4) Total work of penetration=∫FdD

### 2.9. Tablet Ejection Force

Tablet ejection force was measured using the texture analyzer, immediately after compression. The die containing the compressed tablet was placed on a sample holder, and a 9 mm cylindrical probe was adjusted to be at the center of the die. The probe was advanced into the die at the test speed of 10 mm/s. When the trigger force reached 0.1 N, signal recording begun and the probe was consistently advanced at a test speed of 4 mm/s and stopped when the tablet was released from the die. The ejection force was determined by the total probe displacement value (*D*) and the force applied (*F*), using Equation (5).

(5) Tablet ejection force=∫FdD

### 2.10. Tablet Tensile Strength and Porosity

The tablet tensile strength was determined by fracturing the tablet diametrically, on an Ewerka hardness tester at a speed of 0.5 mm/s (Erweka TBH 125, Heusenstamm, Germany). The tensile strength was calculated from the maximum crushing strength, tablet diameter, and tablet thickness, in accordance with Fell and Newton’s method described in Reference [45], in which the tablet tensile strength ($\sigma_{x}$) is represented as follows using the Equation (6),

(6) $$\sigma_{x}=\frac{2x}{\pi dt}$$

where, *x*, *d*, and *t* are the breaking force, tablet diameter, and thickness, respectively.

Tablet dimensions were measured using a micrometer caliper with a precision of 0.01 mm (Mitutoyo, Japan). The true densities of materials were determined using a helium pycnometer (AccuPyc 1330, Micrometrics instruments Co., Norcross, GA, USA). The accuracy of the pycnometer was evaluated using a standard steel sphere before measurements. The experimental sample was accurately weighed and loaded into the sample cell. Sample volume was calculated by measuring pressure, filling the sample chamber with high purity helium gas, followed by discharging the gas into a second empty chamber. The measurements were repeated for five cycles. The percentage tablet porosity (*ε*) was calculated using the Equation (7),

(7) $$\varepsilon \left(\%\right)=\left(1-\frac{D_{tablet}}{D_{true}}\right)\times 100$$

where *ε*, *D_tablet_*, and *D_true_* are the tablet porosity, tablet density, and true density of the formulation, respectively.

## 3. Results and Discussion

### 3.1. Risk Assessment

Quality by design (QbD) is an efficient, risk-controlled, and systematic approach to improve the quality of pharmaceutical products. It can be applied to the entire pharmaceutical production processes or to certain unit operations, and in initial research and development phases [46,47,48]. The International Council for Harmonization of Technical Requirements for Pharmaceuticals for Human Use (ICH) Q8 (R2) guideline considers the quality target product profile (QTPP) an essential element of the QbD approach [49]. It helps to sort out the critical material attributes (CMAs) and critical process parameters (CPPs), that influence critical quality attributes (CQAs). The QTPP for the controlled release EFT is listed in Table 2.

In the present study, the objective of risk assessment was to identify the most important risk factors that needed further investigation. The risk assessment was performed based on the results of screening experiments, prior knowledge, and experience, as well as information regarding effervescent floating systems in previous reports. For the assessments, quantitative risk priority values were mapped into three categories (high, medium, and low), as shown in Table 3. The high-risk factors were introduced as control factors in the experimental design to establish their relationship with response variables, and the low-risk factors were not investigated further, because they have minimal influence on the outcomes.

### 3.2. Polymer Screening

**Figure 1:**
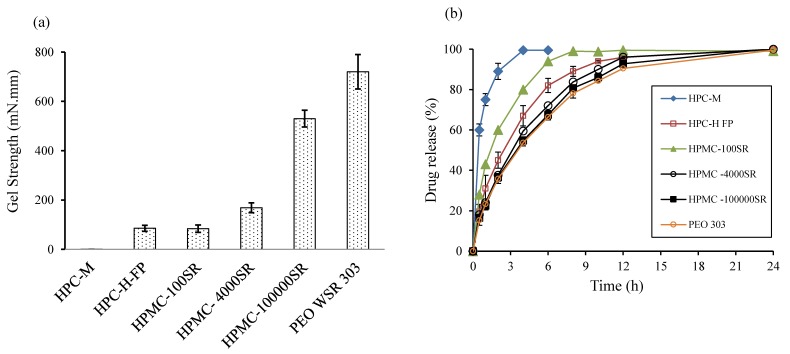
(**a**) Gel strength (mN·mm) of various hydrophilic using the Texture analyzer after being swollen in water at 37 °C. (**b**) Comparison of drug release profiles of the effervescent floating tablets (EFTs) with different hydrophilic polymers.

Various hydrophilic gel-forming polymers were investigated to select the most appropriate one for the metformin EFT, in terms of providing controlled release, high mechanical gel strength, minimum FLT, and high swelling rate. Among the polymers, PEO showed the highest gel strength, whereas HPC M had the lowest strength (Figure 1a). Likewise, among the various viscosity grades of HPMC, HPMC 100,000SR showed the highest gel strength, suggesting that viscosity grade has an impact on the mechanical strength of the gel layer. Higher gel strength may indicate the formation of a stronger gel barrier layer in matrices. Moreover, stronger gel layer of EFT controls the release of the drug, as well as provides mechanical integrity to the matrices [15,41]. However, the tablet might get damaged in the stomach if the outer gel layer is not strong enough and does not resist the external environment. Similarly, EFTs prepared from different polymers suggested that PEO EFT had the lowest FLT and quick penetration of dissolution medium into the matrices (data not shown). 

The in vitro drug release studies of metformin EFT prepared from the polymers are shown in Figure 1b. PEO showed the lowest drug release rate among the studied polymers, whereas HPC M showed the highest rate followed by HPMC 100SR. The decrease in drug release rate might be due to the quick gel-forming capability of PEO, whilst the high release rate from HPC M might be associated with poor hydration rate due to low polymer viscosity. Moreover, in the low viscosity polymers, polymer chains are quickly detangled and the polymer can be eroded eventually leading to rapid drug release [30]. Based on the results, PEO was selected for further optimization studies. 

### 3.3. Evaluation of Medium Uptake, Mass Loss, and Gel Strength

**Figure 2:**
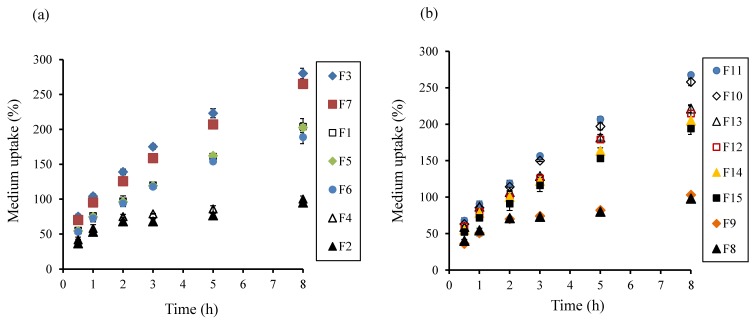
Plots of degree of swelling (medium uptake) vs. time profiles of various formulations based on the Box-Behnken design (BBD) experimental design, (**a**) F1–7 and (**b**) F8–15.

Medium uptake and mass loss were evaluated by comparing the weights of swollen and dried tablets. In the controlled release EFT, hydration capability of the polymer may govern drug release kinetics, as well as tablet buoyancy efficiency [13,26]. As shown in Figure 2a, higher medium uptake was observed in the tablets with higher amounts of PEO, irrespective of compression force in the experimental range. This could suggest that the compression force may not be a critical factor for the medium uptake by PEO. Moreover, the polymer’s capability to absorb the test medium might be due to the presence of hydrophilic groups [50]. Interestingly, at the low level of PEO, sodium bicarbonate did not influence the hydration extent (F2, F4, F8, and F9). However, at the high level, sodium bicarbonate showed a tendency to retard the medium uptake.

**Figure 3:**
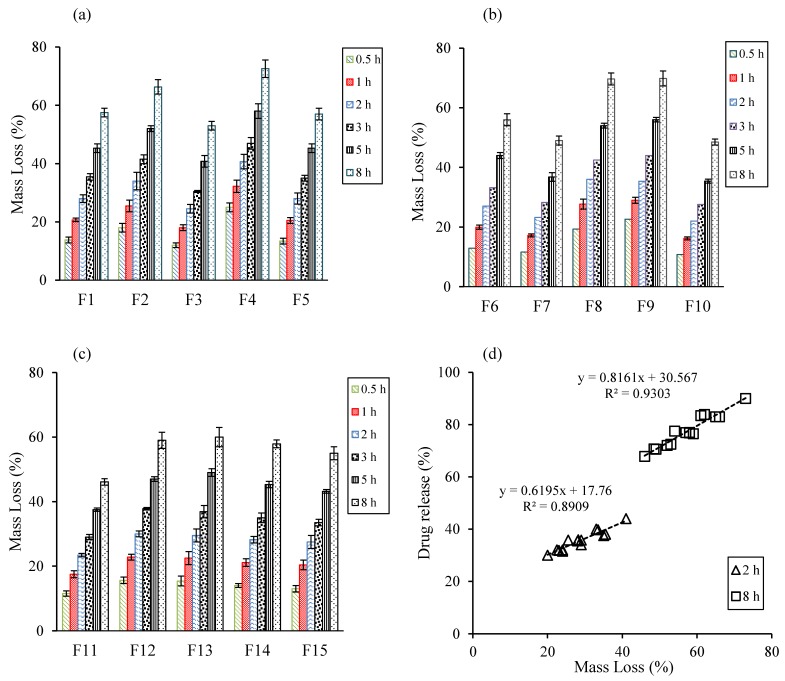
Plots of tablet matrix erosion (% mass loss) vs. time of various formulations based on the BBD experimental design, (**a**) F1–5, (**b**) F6–10, and (**c**) F11–F15). (**d**) Correlation between drug release rate and mass loss in different time points.

Figure 3a–c show the erosion rates of swollen tablets with experimental formulations. Formulations containing higher amounts of PEO (F3, F7, F10, and F11) had low erosion rates, compared to those containing lower amounts of PEO. As shown earlier, high PEO contents in matrices resulted in rapid uptake of dissolution medium and formation of a gel barrier to control drug release. Similarly, formulations containing high amounts of sodium bicarbonate inhibited the erosion rate, which might be attributed to the presence of CO_2_ gas bubbles in the gel layer [17]. Moreover, correlations between the drug release rate and mass loss at 2 h and 8 h, are provided in Figure 3d. The *R*^2^ value at 2 h and 8 h was 0.89 and 0.93, respectively, suggesting high correlation between the drug release rate and mass loss. 

**Figure 4:**
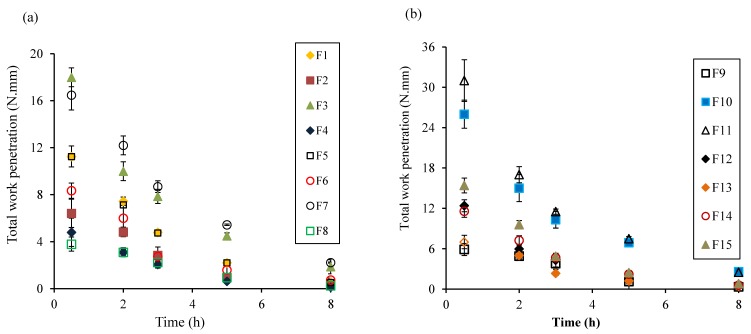
Total work of penetration vs. time profiles of various formulations based on the BBD experimental design, (**a**) F1–8 and (**b**) F9–15.

Textural profiles provide better understanding of the dynamics of gel strength and movement of gel boundaries. To evaluate the gel strength of the swollen tablets, total work of penetration was calculated from the area under the force-displacement curve. As shown in Figure 4, the work of penetration of the tablet sharply decreased with an increase in exposure time due to the increase in the size of polymer molecules, as a consequence of the entry of a medium might decrease the glass transition temperature (*Tg*) [24,51]. As the hydration proceeded, the polymer might have changed from a crystalline state to a rubbery state, and undergone relaxation [15,51]. It was also noticeable that tablets containing higher amounts of PEO (F3, F7, F10, and F11) showed high work of penetration, compared to those with lower PEO amounts. This might be attributed to the formation of a strong gel layer in high PEO matrices.

### 3.4. Evaluation of Drug Release Kinetics

**Figure 5:**
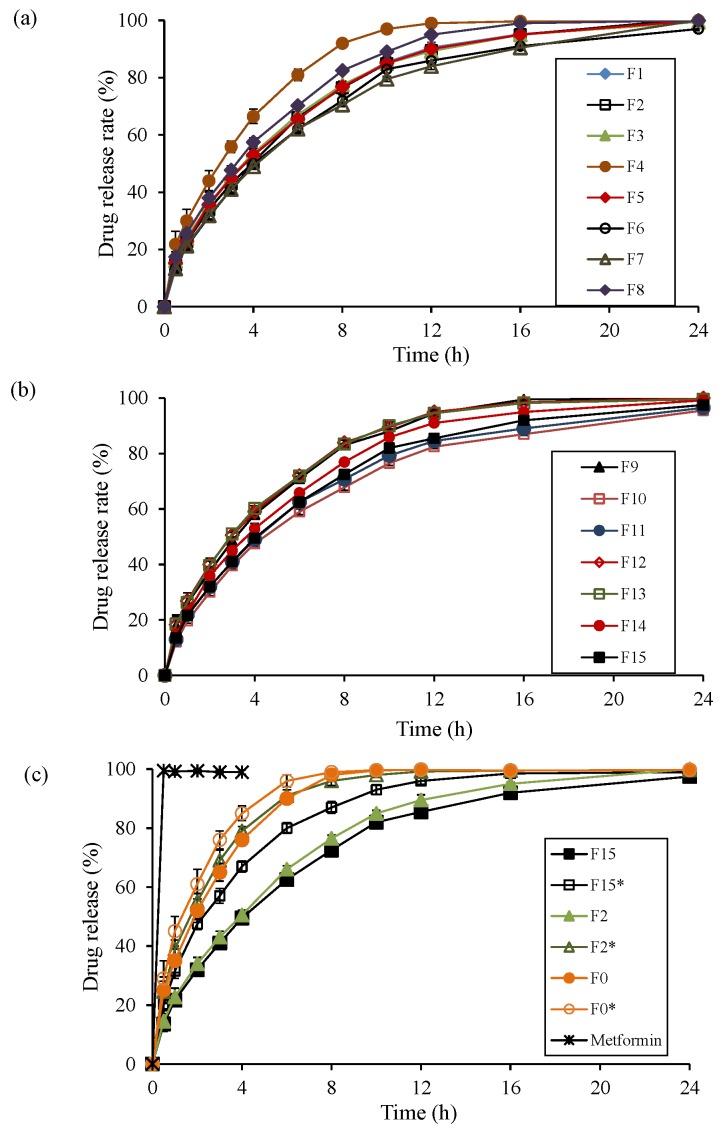
In vitro dissolution profiles of various formulations based on the BBD experimental design, (**a**) F1–8 and (**b**) F9–15. (**c**) Comparison of drug release profiles of tablets with sodium bicarbonate (F0, F2, and F15), and those without sodium bicarbonate (F0*, F2*, and F15*).

In vitro drug release profiles of the experimental tablets (F1–F15), and tablets without sodium bicarbonate (F0*, F2*, and F15*), are provided in Figure 5a–c. Likewise, Table 1 depicts the time required for 25% of drug release (T25%), 50% of drug release (T50%), and 80% of drug release (T80%) in the experimental formulations. Initially (up to 1 h), burst release of metformin was observed in the formulations containing low amounts of polymer and sodium bicarbonate (F4, F8, and F9). This might be ascribed to insufficient time to form a gel barrier at the lower polymer levels, and the high metformin contents in the EFTs. However, with the progression of medium uptake, the polymer might form a gel layer retarding drug molecule transport through the matrix. Moreover, the drug release rate was significantly retarded in formulations containing sodium bicarbonate, compared to the formulations without it (Figure 5c). In the tablets containing sodium bicarbonate, CO_2_ bubbles were liberated extensively when they reacted with the dissolution medium. These CO_2_ bubbles might be entrapped in the gel layer and obstructed diffusion paths, eventually retarding the transport of both drug and dissolution medium through the matrix. This suggests that sodium bicarbonate has a potential role in retarding drug release rates of highly water-soluble drugs in combination with a hydrophilic polymer.

To investigate the release kinetics of metformin EFTs in all the experimental formulations, as well as in F0, F0*, F2*, and F15*, the model equations were fitted to the data of in vitro release profiles [52,53,54,55]: Zero-order equation (Equation (8)), the Korsmeyer-Peppas model (Equations (9)), and the Higuchi model (Equation (10)),

(8) $$M_{t}=M_{0}+k_{0}t$$

(9) $$log(M_{t}/M_{\propto })=\log k+n\log t$$

(10) $$M_{t}=k_{H}\sqrt{t}$$

where *t* is the time, $M_{t}$ is the amount of drug released at time *t*, $M_{0}$ is the initial amount of the drug in solution, $M_{t}/M_{\propto }$ is the fraction of drug released at time *t* (drug loading was considered as $M_{\propto }$), *k*_0_ is the zero-order rate constant, *k* is the Korsmeyer-Peppas rate constant, *n* is the release exponent, and *k_H_* is the Higuchi constant.

As shown in Table 4, correlation coefficients (*R*^2^) in the Korsmeyer Peppas model, the Higuchi model, and the zero-order kinetics equation were determined. Among the models, the Korsmeyer-Peppas model showed the highest linearity, followed by the Higuchi model and the zero-order equation, proposing that in vitro drug release in the formulations was best explained using the Korsmeyer-Peppas model. Furthermore, the *n* value in the Korsmeyer-Peppas equation could be used to explain the drug release mechanism: *n* < 0.5 indicates diffusion transport, whilst *n* value between 0.5–1.0 indicates non-Fickian or anomalous diffusion (i.e., drug release controlled by both diffusion and erosion) [51]. The *n* values of the formulations, including those of F0, F0*, F2*, and F15* were in the range of 0.474–0.701 (Table 4). The formulations except F0, F4, F0*, and F2* showed a non-Fickian or anomalous diffusion, suggesting that drug release was governed by both diffusion and erosion (0.5 < *n* < 1.0). This could be due to the blockade of diffusion by CO_2_ bubbles, as described earlier. However, the formulations of F0, F4, F0*, and F2* showed a diffusion transport (*n* < 0.5). Overall, it could be concluded that formulations containing high levels of both polymer and sodium bicarbonate showed anomalous transport. 

### 3.5. Statistical Analysis and Summary of Fit

A quadratic statistical model, including linear, interactive, and polynomial terms, was used to investigate the influence of control factors on the responses. Table 5 summarizes the coefficients of model terms and associated *p* values for *Y*_1_–*Y*_7_. If the *p* value was less than 0.05 (*p* < 0.05), the factor could be considered to affect the responses significantly. To simplify the regression model, the non-significant terms (*p* > 0.05) were not considered (Equations (11)–(17)). A positive or negative coefficient indicated an increase or decrease in the corresponding response, respectively, to the increase in the level of the factor or factors involved in that term.

To evaluate the validity of the experimental design, analysis of variance (ANOVA), *R*^2^, adjusted *R*^2^, and predicted *R*^2^ were determined (Table 5). The high values of *R*^2^, adjusted *R*^2^, and predicted *R*^2^ indicated good data fitting of the investigated responses. In addition, *p* values of regression models of *Y*_1_–*Y*_7_ were below the significance level (*p* < 0.05), suggesting that the studied response variables were not influenced by any of the control factors. *p* values of lack of fit of *Y*_1_–*Y*_7_ were 0.0508, 0.064, 0.397, 0.108, 0.073, 0.251, and 1.000, respectively, which were greater than 0.05 for all responses, suggesting that model errors were not significant.

### 3.6. Effect of Control Factors on Drug Release Profiles

The actual model *R*^2^, adjusted *R*^2^, and *R*^2^ predicted value for $Y_{1}$ (T25%) were 0.9702, 0.9653, and 0.9493, respectively. Similarly, the actual model *R*^2^, adjusted *R*^2^, and *R*^2^ predicted value for $Y_{2}$ (T50%) were 0.9984, 0.9973, and 0.9910, respectively, and for $Y_{3}$ (T80%) were 0.9980, 0.9972, and 0.9952, respectively. The similarity of these values was suggestive of the goodness of fit. Likewise, the *p*-value of model equation of $Y_{1}$, $Y_{2}$, and $Y_{3}$ were <0.0001. The reduced regression equations in coded terms for $Y_{1}$, $Y_{2}$, and $Y_{3}$, are shown in Equations (11)–(13).

(11) $$Y_{1}=67.66+9.62X_{1}+13.00X_{2}\;$$

(12) $$Y_{2}=229.000000+32.000000X_{1}+X_{2}36.750000-0.000003X_{3}-9.500040X_{1}X_{2}\;-8.000020X_{2}^{2}-2.999980X_{3}^{2}$$

(13) $$Y_{3}=527.08+72.38X_{1}+64.00X_{2}+9.12X_{1}^{2}-14.13X_{2}^{2}$$

As shown in Table 5, the amount of PEO ($X_{1}$) and sodium bicarbonate ($X_{2}$) had a significant effect on $Y_{1}$, $Y_{2}$, and $Y_{3}$. Coefficients of $X_{1}$ and $X_{2}$ were positive for $Y_{1}-Y_{3}$, suggesting that the dependent variables increased with the increase of PEO and sodium bicarbonate. In addition, coefficients of $X_{1}^{2}$ for $Y_{3}$ were positive, suggesting a synergistic effect on drug release response, whilst negative coefficients of $X_{2}^{2}$ for $Y_{2}$ and $Y_{3}\;$indicated an antagonist effect on drug release response. Furthermore, in case of $Y_{2}$ and $Y_{3}$, the coefficient of $X_{1}X_{2}$ had a negative effect, which indicated that the $X_{1}X_{2}$ interaction term had a reciprocal relation with $Y_{2}$ and $Y_{3}$. This can be explained on the basis that as the amount of sodium bicarbonate increased in the tablet, more CO_2_ was generated, which was entrapped in the gel layer and obstructed diffusion, thereby reducing drug transport through the matrix. In the previous studies, polymer content in the matrix was highlighted as an important variable in controlling release rate [10,13,38]. However, in case of highly water-soluble drugs loaded at high levels, a large amount of polymer is required in the formulation to extend release rate, which often poses challenges for formulation scientists [56,57]. However, our finding suggests that sodium bicarbonate could contribute to controlling the release rate of highly water-soluble drugs, and potentially reduce the polymer amount in the formulation.

**Figure 6:**
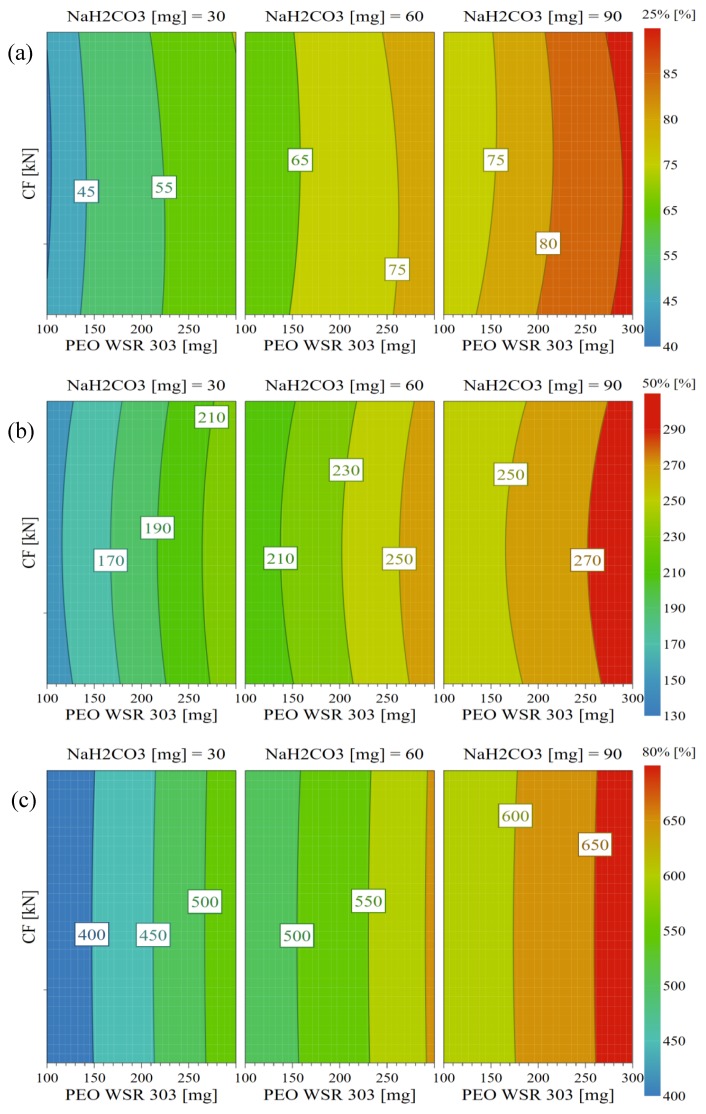
Contour plots showing the effects of hydrophilic polymer (PEO) and sodium bicarbonate on the drug release responses (*Y*_1_*–Y*_3_): (**a**) T25% (*Y*_1_), (**b**) T50% (*Y*_2_), and (**c**) T80% (*Y*_3_).

In addition, a contour plot (Figure 6) was used to visualize the influence of tablet compression force and the concentrations of PEO and sodium bicarbonate on *Y*_1_, *Y*_2_, and *Y*_3_. The plot showed that the time required for drug release from the EFT, significantly increased with the sodium bicarbonate level. This might be attributed to the increased release of CO_2_ bubbles at the high levels of sodium bicarbonate, when reacted with the dissolution medium. As suggested earlier, the liberated CO_2_ bubbles may interfere the transport of drug and water through the matrix. Likewise, at a constant sodium bicarbonate level, an increase in PEO concentration increased *Y*_1_, *Y*_2_, and *Y*_3_. Increase in PEO concentration may cause the dissolution medium to penetrate quick into the tablet and form a thick gel layer. As a result, the diffusion path length would be increased, retarding the drug release rate. In addition, at high PEO levels, PEO tortuosity might increase, facilitating entanglement of the polymer chains [56,58].

### 3.7. Effect of Control Factors on Floating Lag Time (FLT)

FLT is the time required for the tablet to float on the surface of dissolution medium, after its introduction into the medium. Dosage form density might be associated with floating behavior. Previous studies showed that tablets with density greater than 1.004 g/cm^3^ could not float on gastric fluid [3,59]. In the floating system, a shorter FLT is preferable. It is generally assumed that as FLT increases, the tablet may attach to the lower part of the stomach and be unable to float, leading to an increase in the chances of gastric emptying. Therefore, FLT may be an important factor affecting gastric retention time, requiring minimization. 

**Figure 7:**
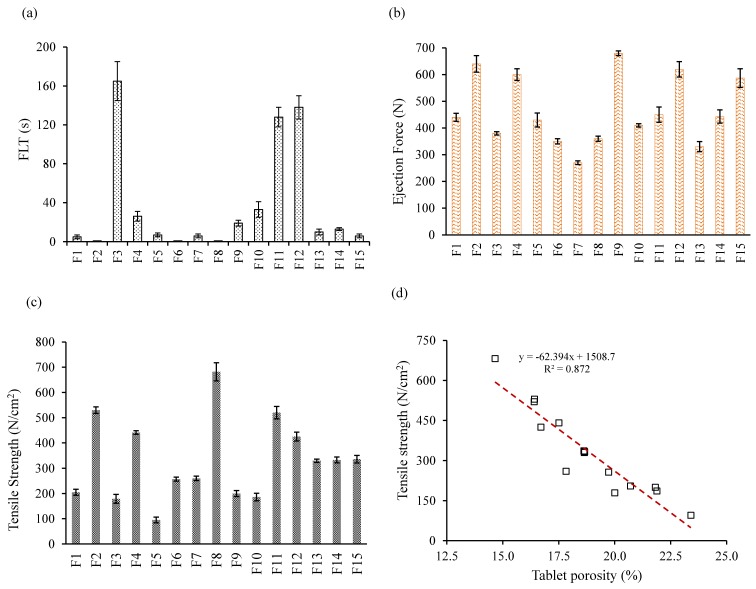
Evaluation of tablet properties of the BBD experimental formulations (**a**) floating lag time (FLT (s)), (**b**) tablet ejection force (N), (**c**) tablet tensile strength (N/cm^2^), and (**d**) correlation between tablet tensile strength and tablet porosity (%).

Figure 7a shows the FLT values of the experimental formulations. The actual model *R*^2^, adjusted *R*^2^, and *R*^2^ predicted, for FLT ($Y_{4}$) were 0.9859, 0.9670, and 0.8668, respectively, and were close to 1. The similarity of these values was suggestive of the goodness of fit. The reduced regression equations in coded terms for FLT $Y_{1}$, are shown in Equation (14).

(14) $$Y_{4}=12.23+35.63X_{1}-37.25X_{2}+34.12X_{3}-26.75X_{1}X_{2}+26.00X_{1}X_{3}-30.75X_{2}X_{3}\;+23.35X_{1}^{2}+23.60X_{2}^{2}\;$$

As shown in Table 5, linear, interaction, and polynomial terms of the control factors had a significant influence on the FLT. The coefficients of $X_{1}$,$\;X_{3}$, $X_{1}X_{3}$, $X_{1}^{2}$, and $X_{2}^{2}$ were positive, whilst coefficients of $X_{2}$, $X_{1}X_{2}$, and$\;X_{2}X_{3}$ were negative (Equation (14)). This suggested that FLT increased with the increasing amount of PEO and/or compression force but decreased with the increase in sodium bicarbonate.

**Figure 8:**
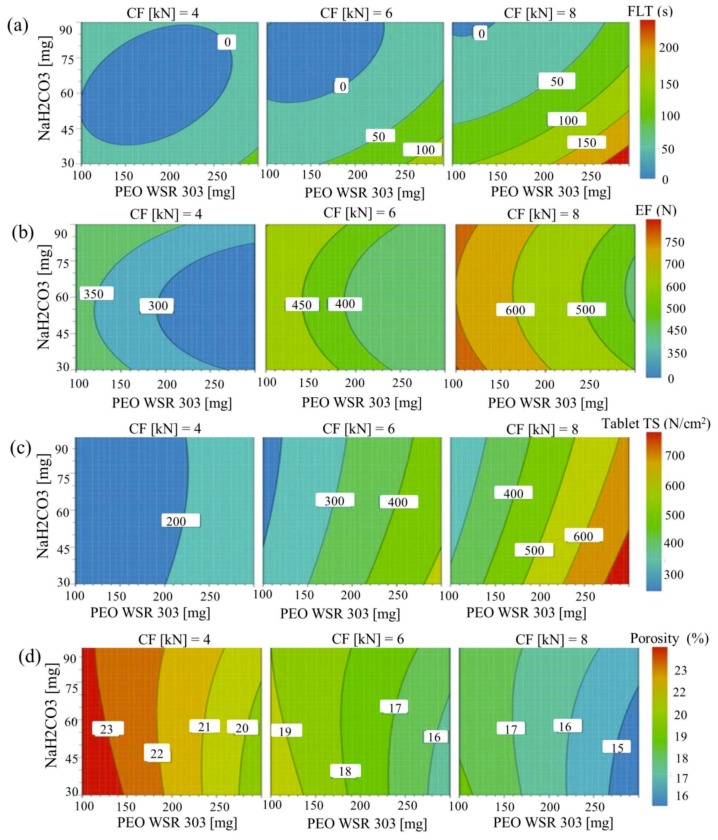
Contour plots showing the effects of hydrophilic polymer (PEO) and sodium bicarbonate on (**a**) floating lag time, (*Y*_4_), (**b**) tablet ejection force, (*Y*_5_), (**c**) tablet tensile strength, (*Y*_6_), and (**d**) tablet porosity (*Y*_7_).

Figure 8a shows the influence of PEO and sodium bicarbonate on the FLT at low, moderate, and high level of compression force. FLT increased significantly with an increase in the compression force. As compression force increased, tablet density would increase because of decreased tablet porosity. To float on the surface of the gastric fluid, tablet density would need to be less than that of the gastric fluid. At all compression force values, increase in the amount of sodium bicarbonate resulted in a reduction in FLT, whilst an increase in the PEO amount led to an increase in FLT; a high level of sodium bicarbonate may lower tablet density by quickly releasing CO_2_ upon contact with the dissolution medium. However, increase in PEO could reduce tablet porosity and increase tablet density, increasing the FLT. Furthermore, at a higher PEO level, an outer gel layer may form quickly upon contact with the simulated gastric fluid, and retard its exposure to sodium bicarbonate, further delaying the FLT.

### 3.8. Effect of Control Factors on Tablet Properties

Process development and formulation design of tablet dosage forms, need a thorough understanding of physicochemical properties and the deformation nature of API and excipients. These properties have a significant impact on compaction behavior during tableting. During the tablet compression, various factors, such as mechanical interlocking, solid bridging, particle fragmentation, and van der Waals’ forces, contribute to packing and bonding of pharmaceutical materials [60]. Tablet compression involves several stages, including particle rearrangement, deformation, fragmentation, decompression, and ejection. A high tablet ejection force may indicate a high frictional force at the tablet-die wall interface, which could damage the tablet and reduce tooling life due to wear [61]. In general, brittle materials produce tablets with a rough surface increasing the frictional force at the interface of the die wall and tablet, eventually increasing tablet ejection force. Even though lubricants can be used to reduce the frictional force during tablet compression, use of a lubricant in excessive amounts in tablets could have adverse effects on tablet quality, including low tablet tensile strength [62], increased tablet friability [63], and reduced dissolution rate [64]. One approach of reducing tablet ejection force is the use of both brittle and plastic deforming materials in the formulation, as the deforming nature of powder including brittle, plastic, and elastic deforming could influence tablet properties, such as ejection force, tensile strength, and porosity [32,39]. 

#### 3.8.1. Effects of Control Factors on Tablet Ejection Force

Tablet ejection force of different experimental runs ranged from 270 to 680 N, as shown in Figure 7b. A regression equation with control factors affecting the ejection force used to generate the empirical model is described by Equation (15)

(15) $$Y_{5}=440.71-91.87X_{1}+4.00X_{2}+127.13X_{3}-37.5X_{1}X_{3}+48.29X_{2}^{2}$$

As shown in Table 5, *p* value < 0.05 for any of the factors, represents a significant effect of the corresponding factors on tablet ejection force. The coefficients of $X_{2}$, $X_{3}$, and $X_{2}^{2}$ were positive, while the coefficients of $X_{1}$ and $X_{1}X_{3}$ were negative, suggesting that tablet ejection force decreased with the increase in PEO level and increased with the increase in compression force. The actual model *R*^2^, adjusted *R*^2^, and *R*^2^ predicted, for tablet ejection force ($Y_{5}$) were 0.9747, 0.9607, and 0.9135, respectively. The similarity of these values was suggestive of the goodness of fit.

Effects of PEO and sodium bicarbonate amounts on tablet ejection force at low, moderate, and high compression force, are shown in a contour plot (Figure 8b). As the compression force increased from 4 to 8 kN, ejection force significantly increased. As the ejection force increased, frictional force at the tablet-die wall interface would increase, eventually increasing the tablet ejection force. At constant compression force and PEO, tablet ejection force decreased at a low sodium bicarbonate level, but increased at a moderate level, suggesting a quadratic effect of sodium bicarbonate (nonlinear relation). However, this effect was not significant (*p* = 0.62). Moreover, at constant ejection force and sodium bicarbonate, an increase in PEO amount resulted in a decrease in tablet ejection force. This might be attributed to the plastic deforming nature of PEO, resulting in a low frictional force at the tablet-die wall interface [32,65]. In addition, decreasing PEO amount in the tablet resulted in an increase in the amount of lactose monohydrate (used for tablet weight adjustment). As lactose monohydrate is a brittle deforming excipient, increasing its amount in the tablet could have resulted in a high frictional force at the tablet-die wall interface, eventually increasing ejection force.

#### 3.8.2. Effect of Control Factors on Tablet Tensile Strength

Mechanical strength of tablets is crucial in controlled release formulations, since tablets with low mechanical strength could have poor friability causing breakage, and therefore may be unable to resist stresses during downstream processing, including tablet coating, packaging, and shipping. Therefore, to ensure sufficient mechanical strength, tablet tensile strength was assessed. Tablet tensile strength of the experimental formulations ranged from 95 to 690 N (Figure 7c). The actual model *R*^2^, adjusted *R*^2^, and *R*^2^ predicted, for tablet tensile strength ($Y_{6}$) were 0.9978, 0.9962, and 0.9896, respectively. The similarity of these values was suggestive of the goodness of fit. The reduced regression equations in coded terms for $Y_{6}$, are shown in Equation (16).

(16) $$Y_{6}=332.73+148.75X_{1}-24.50X_{2}+141.75X_{3}-13.75X_{1}X_{2}+62.25X_{1}X_{3}-15.25X_{2}X_{3}\;$$

As shown in Table 5, *p* value < 0.05 of any of the factors, represents a significant effect of the corresponding factors on the tensile strength. It was observed using Equation (16), that the coefficients of $X_{1}$,$\;X_{3}$, and $X_{1}X_{3}$ were positive, while the coefficients of $X_{2}$, $X_{1}X_{2}$, and $X_{2}X_{3}$ were negative. This suggested that PEO content and compression force had a synergistic effect on the tablet tensile strength, whilst sodium bicarbonate amount had an antagonistic effect. Similarly, the effect of PEO and sodium bicarbonate levels on the tensile strength at low, moderate, and high compression force is provided in a contour plot (Figure 8c). At low compression force, increasing the amount of PEO contributed to a slight increase in the tensile strength. However, at medium and high compression force, increase in PEO levels resulted in a drastic increase in the tensile strength. This may be attributed to high mechanical interlocking, van der Waal’s forces, and solid bridging between the particles at high compression force. In addition, the more ductile behavior of PEO may allow plastic deformation during tableting, yielding stronger tablets. At constant compression force and PEO, an increase in the concentration of sodium bicarbonate resulted in a slight reduction in the tablet tensile strength. This may be due to the poor compaction tendency of sodium bicarbonate. 

#### 3.8.3. Effect of Control Factors on Tablet Porosity

Tablet porosity of the experimental formulations ranged from 14.7% to 23.4%. The actual model *R*^2^, adjusted *R*^2^, and *R*^2^ predicted, for tablet tensile strength ($Y_{6}$) were 0.9999, 0.9998, and 0.9998, respectively. The similarities of these values suggest the goodness of fit. The reduced regression equation in coded terms for $Y_{7}$ is shown in Equation (17).

(17) $$Y_{7}=18.63-1.70X_{1}+0.10X_{2}-2.65X_{3}+0.44X_{1}X_{2}+0.15X_{1}X_{3}+0.05X_{2}X_{3}-0.14X_{1}^{2}+\;0.15X_{2}^{2}+0.40X_{3}^{2}\;$$

As shown in Table 5, *p* value < 0.05 of any of the factors, represented a significant effect of the corresponding factors on the tablet porosity. Compression force showed the most significant effect on the porosity among the studied variables. The coefficients of $X_{1}$,$\;X_{3}$, and $X_{1}^{2}$ were negative, while the coefficients of $X_{2}$, $X_{1}X_{2}$, $X_{2}X_{3}$, $X_{1}X_{3}$, $X_{2}^{2}$, and $X_{3}^{2}$ were positive. This suggested that the amount of PEO and compression force were inversely proportional to the porosity, whilst the amount of sodium bicarbonate was directly proportional to the porosity.

A contour plot (Figure 8d) showed the effect of PEO and sodium bicarbonate levels on tablet porosity at low, moderate, and high compression force. As expected, tablet porosity decreased significantly with the increase of compression force. At high levels of PEO and compression force, void space between the particles reduced drastically due to high mechanical interlocking and bonding forces between the particles. Moreover, PEO was highly compressible due to its plastic deforming nature, forming strong solid bridging between particles. In contrast, slightly higher porosities were found at higher sodium bicarbonate amounts due to its poor compressibility. Moreover, tablet porosity was highly correlated with tablet tensile strength, as shown in Figure 7d, suggesting that tablet tensile strength decreased with the increase in tablet porosity. Based on the experimental data, a regression model between tablet porosity and tensile strength can be obtained as y=−62.39x+1508.7, suggesting that tablet porosity higher than 20% produces tablets with low tensile strength. 

### 3.9. Design Space and Optimization

**Figure 9:**
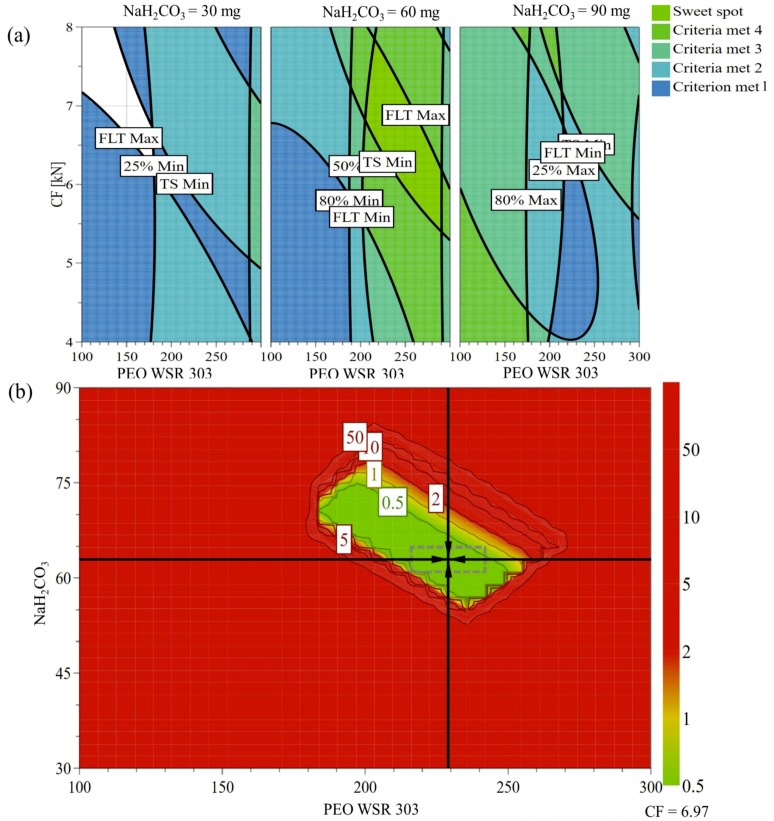
(**a**) Sweet spot plots of PEO amount (*x*_1_) and sodium bicarbonate (*x*_2_) at low (**left**), medium (**middle**), and high (**right**), and compression force *(x*_3_), defined in the specification of time required for release: 25% (50 ≤ *Y*_1_ ≤ 80 min; target, 60 min); 50% (220 ≤ *Y*_2_ ≤ 260 min; target, 240 min); and 80% (510 ≤ *Y*_3_ ≤ 570 min; target, 540 min). FLT (1 ≤ *Y*_4_ ≤ 60 s; target, 30 s), and tensile strength (TS) (350 ≤ *Y*_6_ ≤ 670 N/cm^2^; target, 500 N/cm^2^). (**b**) Design space in terms of PEO amount (*x*_1_) and sodium bicarbonate (*x*_2_) at low (**left**), medium (**middle**), and high (**right**), and compression force *(x*_3_), defined in the specification of time required for release after the Monte Carlo simulation.

Design space (DS) is the multidimensional space of formulation and process setting, where the predetermined product quality attributes remain within the specification, when formulation and/or process variables are being changed [66]. To identify the design space, the knowledge space is divided into smaller subspaces, and the probability of fulfilling the specification within each region is evaluated. In the present study, design space was generated using a Monte Carlo simulation. The desired specification in the current investigation was to provide the controlled drug release rate, as well as the tablet buoyancy for 12 h. Therefore, the design space was explored based on the desired targets for the drug release rate; T25 (50 ≤ *Y*_1_ ≤ 80 min; target, 60 min); T50 (220 ≤ *Y*_2_ ≤ 260 min; target, 240 min); T80 (510 ≤ *Y*_3_ ≤ 570 min; target, 540 min); FLT (1 ≤ *Y*_4_ ≤ 60 s; target, 20 s); and tablet TS (350 ≤ *Y*_6_ ≤ 670 N/cm^2^; target, 500 N/cm^2^). The resulting design space is provided in Figure 9a. Furthermore, a sweet plot was also constructed to explain the influence of control factors on the response variables. The plots were designed based on the given specifications of drug release rate, FLT, and tablet tensile strength (Figure 9b). The green region denoted in the color index represents fulfillment of all criteria, suggesting the appropriate region to obtain the desired outputs (Figure 9b).

Even though design space represents the region of theoretical robustness, experimental robustness testing provides validation of the design. To obtain a robust point close to the selected optimal point, robustness testing can be performed. The identified robust point was characterized by the combination of control factors comprising 221 mg PEO per tablet, 62 mg sodium bicarbonate per tablet, and 7 kN tablet compression force. The obtained experimental robust points were 65 min, 237 min, 548 min, 32 s, and 450 N/cm^2^ for *Y*_1_, *Y*_2_, *Y*_3_, *Y*_4_, and *Y*_6_, respectively.

## 4. Conclusions

Polymers with a high viscosity grade and molecular weight provided high mechanical gel strength, and retarded drug release rate. Our results showed that PEO contributed significantly to controlling drug release, improving gel strength, and improved tablet properties. The results also provided evidence that sodium bicarbonate had a dual function in highly water-soluble drug EFTs, i.e., it improved tablet floating and enabled controlled release by retarding the drug release rate from the hydrophilic matrices. The various factors tested were negatively correlated with the tablet properties. Interestingly, PEO sharply reduced tablet ejection force, which could improve the tablet preparation process. Overall, the present study provided a perspective on systematically fabricating EFTs loaded with high doses of highly water-soluble drugs by applying design space, and quality by design concepts.

## Figures and Tables

**Table 1:** Box Behnken experimental design with three independent variables (control factors), and dependent variables of drug release at different time points, floating lag time, tablet ejection force, tablet tensile strength, and tablet porosity.

Run	Independent Variables			Dependent Variables						
	*X*_1_	*X*_2_	*X*_3_	*Y*_1_	*Y*_2_	*Y*_3_	*Y*_4_	*Y*_5_	*Y*_6_	*Y*_7_
	PEO WSR (mg)	Sodium Bicarbonate (mg)	Compression Force (kN)	T25% (min)	T50% (min)	T80% (min)	FLT (s)	Ejection Force (N)	Tensile Strength (N/cm^2^)	Tablet Porosity (%)
1	200	60	6	70	229	528	5	445	342	18.7
2	100	90	6	72	238	516	1	625	179	20.0
3	300	30	6	61	224	529	165	390	524	16.4
4	100	30	6	44	143	391	26	600	207	20.7
5	200	60	6	69	228	531	7	430	330	18.6
6	200	90	4	79	254	580	1	350	188	21.9
7	300	60	4	80	260	612	6	280	271	19.7
8	100	60	4	59	193	461	1	360	90	23.4
9	100	60	8	57	194	459	19	680	260	17.8
10	300	90	6	87	281	655	33	410	441	17.5
11	300	60	8	81	259	610	128	450	690	14.7
12	200	30	8	55	183	444	138	620	503	16.4
13	200	30	4	53	181	450	10	330	200	21.8
14	200	60	6	69	228	525	13	440	336	18.6
15	200	90	8	79	252	575	6	587	430	16.7

**Table 2:** Quality target product profiles (QTPP) of effervescent floating tablets.

QTPP		Target	Justification
Dosage form		Controlled release effervescent floating tablet	Metformin is well absorbed from the upper part of the small intestine Short half-life and highly water-soluble
Route of administration		Oral	Designed for oral administration
Dose strength		500 mg/Tablet	Commonly accepted strength
Product quality attributes	Tablet TS	500 N/cm^2^	Softer tablet could chip or break during packaging and/or shipping, unacceptable for controlled release formulations. High tablet hardness can increase FLT.
	Assay	100% *w*/*w* of label claim	Assay variability will affect safety and efficacy. Process variables could affect product assay.
	Dissolution	Sustained release up to 12 h	Failure to meet dissolution specifications can impact bioavailability. Amount of polymer and effervescence will impact drug release rate.
	FLT	As low as possible	Prevent gastric emptying
	Floating time	12 h	Tablet should continuously float in medium. If not, gastric emptying of the tablet is likely to occur, which can be considered failure.
	Tablet ejection force	As low as possible	Minimize tablet defects and improve product quality and production output.

**Table 3:** Initial risk assessment for formulation and process variables in controlled release effervescent floating tablets.

Drug Product CQAs	Formulation Variables					Process Variables	
	Type/Viscosity Grade of Polymer	PEO WSR 303	Sodium Bicarbonate	MCC	Lactose Monohydrate	Compression Force	Dwell Time
Drug release: T25%, T50% and T80%	High	High	High	Low	Low	Low	Low
Assay	Low	Low	Low	Low	Low	Low	Low
Floating lag time	Medium	Medium	High	Medium	Low	High	Medium
Tensile strength	High	High	Low	Medium	Low	High	Medium
Gel strength	High	High	Low	Low	Low	Medium	Low
Ejection force	High	High	Low	Medium	Medium	High	Low

Low: Broadly acceptable risk. No further investigation needed; Medium: Risk acceptable. Further investigation may need to reduce the risk; High: Risk unacceptable. Further investigation needed to reduce the risk.

**Table 4:** Correlation coefficient (*R*^2^) values for the dissolution profiles plugged into various release models.

Run	Zero-Order	Higuchi	Korsmeyer-Peppas		
	*R* ^2^	*R* ^2^	*R* ^2^	*n*	*k*
F1	0.9355	0.9970	0.9999	0.575	0.375
F2	0.9413	0.9959	0.9991	0.599	0.234
F3	0.9229	0.9957	0.9996	0.567	0.376
F4	0.8871	0.9979	0.9876	0.490	0.633
F5	0.9401	0.9977	0.9995	0.584	0.370
F6	0.9469	0.9944	0.9986	0.639	0.158
F7	0.9408	0.9962	0.9990	0.612	0.210
F8	0.9222	0.9961	0.9987	0.534	0.478
F9	0.9238	0.9959	0.9969	0.535	0.488
F10	0.9450	0.9940	0.9937	0.702	0.100
F11	0.9415	0.9956	0.9987	0.632	0.210
F12	0.9132	0.9945	0.9983	0.554	0.450
F13	0.9167	0.9934	0.9984	0.541	0.452
F14	0.9307	0.9969	0.9983	0.577	0.373
F15	0.9475	0.9946	0.9945	0.641	0.160
F0	0.7702	0.9936	0.9675	0.485	0.730
F0*	0.8333	0.9985	0.9825	0.474	0.792
F2*	0.8174	0.9947	0.9740	0.481	0.744
F15*	0.8787	0.9937	0.9838	0.512	0.600

**Table 5:** Regression coefficients and associated *p* values for the responses.

Terms	T25%		T50%		T80%		FLT		Tablet Ejection Force		Tablet Tensile Strength		Tablet Porosity	
	(*Y*_1_)		(*Y*_2_)		(*Y*_3_)		(*Y*_4_)		(*Y*_5_)		(*Y*_6_)		(*Y*_7_)	
	Coefficient	*p* Value	Coefficient	*p* Value	Coefficient	*p* Value	Coefficient	*p* Value	Coefficient	*p* Value	Coefficient	*p* Value	Coefficient	*p* Value
Constant	67.667	<0.0001	229.000	<0.0001	527.077	<0.0001	12.230	0.04712	440.714	<0.0001	332.733	<0.0001	18.633	<0.0001
*X*_1_	9.625	<0.0001	32.000	<0.0001	72.375	<0.0001	35.625	<0.0001	−91.875	<0.0001	148.750	<0.0001	−1.700	<0.0001
*X*_2_	13.000	<0.0001	36.750	<0.0001	64.000	<0.0001	−37.250	<0.0001	4.0000	0.6248	−24.500	0.0001	0.1000	0.001
*X*_3_	–	–	0.0000	1.0000	–	–	34.125	<0.0001	127.1200	<0.0001	141.750	<0.0001	−2.650	<0.0001
*X*_1_ *X_2_	–	–	−9.500	<0.0001	–	–	26.750	0.0019	–	–	−13.750	0.0239	−0.450	<0.0001
*X*_2_ *X_3_	–	–	–	–	–	–	−30.750	0.0010	–	–	−15.250	0.0150	0.050	0.0408
*X*_1_ *X_3_	–	–	–	–	–	–	26.000	0.0022	−37.5000	0.0140	62.250	<0.0001	0.150	0.0004
*X*_1_^2^	–	–	–	–	9.115	<0.0001	23.346	0.0045	–	–	9.5000	0.0010	−0.141	<0.0001
*X*_2_^2^	–	–	−8.000	<0.0001	−14.134	0.0011	23.596	0.0043	48.2856	0.0043	–	–	0.158	<0.0001
*X*_3_^2^	–	–	−3.000	0.0180	–	–	–	–	–	–	–	–	0.408	<0.0001
*R* ^2^	0.9702		0.9984		0.9980		0.9859		0.9747		0.9978		0.9999	
*R*^2^ (Adj)	0.9653		0.9973		0.9972		0.9670		0.96.07		0.9962		0.9998	
*R*^2^ (Pred)	0.9493		0.9910		0.9952		0.8668		0.9135		0.9896		0.9998	

– indicates the insignificant term (*p* > 0.05).
